# Notch Signaling Regulation in HCC: From Hepatitis Virus to Non-Coding RNAs

**DOI:** 10.3390/cells10030521

**Published:** 2021-03-01

**Authors:** Catia Giovannini, Francesca Fornari, Fabio Piscaglia, Laura Gramantieri

**Affiliations:** 1 Department of Experimental, Diagnostic and Specialty Medicine (DIMES), University of Bologna, 40138 Bologna, Italy; 2 Center for Applied Biomedical Research (CRBA), S.Orsola-Malpighi University Hospital, 40138 Bologna, Italy; francesca.fornari2@unibo.it; 3 Department for Life Quality Studies, University of Bologna, 47921 Rimini, Italy; 4 Division of Internal Medicine, IRCCS Azienda Ospedaliero-Universitaria di Bologna, 40138 Bologna, Italy; fabio.piscaglia@unibo.it (F.P.); laura.gramantieri@aosp.bo.it (L.G.); 5 Department of Medical and Surgical Science (DIMEC), University of Bologna, 40138 Bologna, Italy

**Keywords:** NOTCH, Hepatocellular carcinoma, ncRNAs, cancer therapy

## Abstract

The Notch family includes evolutionary conserved genes that encode for single-pass transmembrane receptors involved in stem cell maintenance, development and cell fate determination of many cell lineages. Upon activation by different ligands, and depending on the cell type, Notch signaling plays pleomorphic roles in hepatocellular carcinoma (HCC) affecting neoplastic growth, invasion capability and stem like properties. A specific knowledge of the deregulated expression of each Notch receptor and ligand, coupled with resultant phenotypic changes, is still lacking in HCC. Therefore, while interfering with Notch signaling might represent a promising therapeutic approach, the complexity of Notch/ligands interactions and the variable consequences of their modulations raises concerns when performed in undefined molecular background. The gamma-secretase inhibitors (GSIs), representing the most utilized approach for Notch inhibition in clinical trials, are characterized by important adverse effects due to the non-specific nature of GSIs themselves and to the lack of molecular criteria guiding patient selection. In this review, we briefly summarize the mechanisms involved in Notch pathway activation in HCC supporting the development of alternatives to the γ-secretase pan-inhibitor for HCC therapy.

## 1. Introduction

### Hepatocellular Carcinoma

Hepatocellular carcinoma (HCC) accounts for 80–90% of liver cancers and represents the third leading cause of cancer mortality worldwide. Over the past two decades, the incidence of HCC has doubled in many countries including Europe and United States [1]. While approximately 75% of HCCs were associated with hepatitis B or C infection, other major risk factors including aflatoxin B1 (AFB1) exposure, nonalcoholic fatty liver disease (NAFLD), chronic alcohol consumption and obesity are now gaining more and more relevance [2]. Among treatment options, surgical resection and percutaneous ablation are considered “curative” modalities; however, they can be performed in a restricted fraction of patients, particularly those detected at early stages. When diagnosed at, or progressed to, intermediate-stage, the standard of care is transarterial chemoembolization [3], while large tumors or HCC with vascular invasion can be considered for radioembolization with selective internal radiation treatment (SIRT). When diagnosed at advanced stages or in the presence of extra-hepatic spread, only systemic treatments can be administered. Sorafenib, the first oral multikinase inhibitor (MKI) entering the treatment of HCC, has been the standard of care for almost ten years [4]. It inhibits angiogenesis and cell proliferation by targeting platelet-derived growth factor receptor (PDGF-R), vascular endothelial growth factor receptor (VEGFR-2/3), c-Kit, Flt3 and Raf kinases involved in the MAPK/ERK pathway [5].The second tyrosine kinase inhibitor to be approved for the first line treatment of advanced HCC is lenvatinib, based on its non-inferiority to sorafenib [6]. In the setting of second line treatments, regorafenib and cabozantinib were approved for patients with HCC progression and ramucirumab for patients previously treated with sorafenib, with a serum AFP (Alpha-Fetoprotein) level ≥400 ng/mL [7]. Beside these molecularly-targeted agents, immuno-oncology has opened the way to a novel class of drugs modulating the expression of Immune Check Point Inhibitors (ICPI), whose aberrant expression results in the immune escape of cancer cells [8]. Programmed cell death protein 1 (PD1), Programmed Death-Ligand 1 (PDL1), Cytotoxic T-Lymphocyte-Associated Protein 4 (CTLA4) are the most targeted ICPI in HCC proving to be more effective than Tyrosine Kinase Inhibitors (TKI), especially in combination regimens [9]. The most interesting results have been obtained so far with the association of Atezolizumab and Bevacizumab [10], however other promising combinations are under clinical investigation. Large clinical trials are however still needed for validation of these effects and for the identification of biomarkers helping the allocation of patients to the most effective treatment option in a personalized perspective. In addition, beside the fraction of patients who are non-responders to ICPIs, others may experience adverse events which prevent the prosecution of treatment. Unfortunately, the molecular classification of HCC has not entered clinical practice so far; thus, genetic and epigenetic factors driving response or resistance to each treatment are still poorly understood.

Deregulation of multiple molecular signaling pathways such as Wnt/β-catenin, Ras mitogen-activated protein kinase (Ras/Raf/MAPK), phosphatidylinositol 3-kinase (PI3K), AKT/mammalian target of rapamycin (mTOR), Janus kinase (Jak)-signal transducer activator of transcription factor (Stat) (JAK/STAT) and the Hippo signaling pathway are essential for HCC development and progression [11]. Understanding the critical genes and signaling molecules in different HCC subgroups will help to develop tailored therapeutic strategies. Inhibition of a single signaling cascade may induce feedback activation of other pathways; hence, combination of different molecularly targeted agents is expected to show synergistic activity. Notch signaling modulates the development and functions of several immune cell lineages. Among these, Notch1 and Dll4 interaction participates in T cells’ lineage commitment, while Notch2-Dll1 interaction contributes to the development of marginal zone B cells [12]. In the peripheral T and B cell compartment, Notch signaling activates T cells’ proliferation and cytokine production, which, in line, are down-regulated by GSI-mediated inhibition of Notch. Similarly, Notch1 and Notch2 activation promotes naive CD8+ T cells to cytotoxic T lymphocytes, which, in turn, drive the antitumoral response and are the targets of ICPIs.

Investigations on Notch signaling in T cells functions might lead to a more effective avenue for combined intervention in cancer treatment.

The aim of this review is to discuss recent advances in molecular mechanisms involved in Notch signaling regulation that could represent new challenges for HCC therapy. Studies described in this review might contribute to the identification of key molecules responsible for Notch pathway regulation that could represent new therapeutic targets.

## 2. Notch Genes

**Figure 1:**
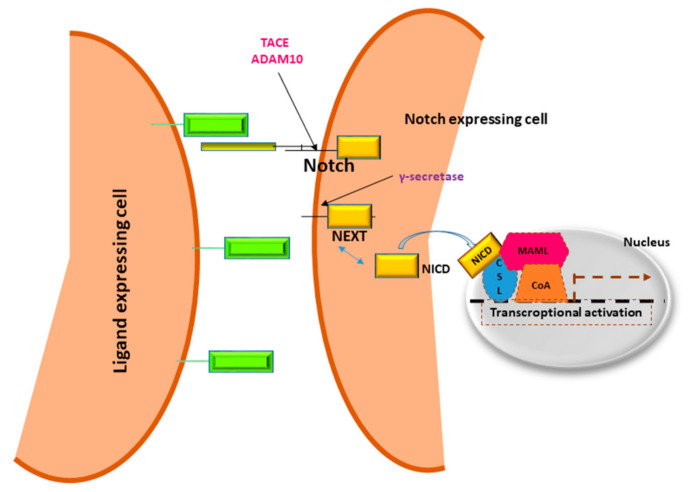
Notch signaling pathway.

In 1911, Morgan and colleagues described a *Drosophila* with a notch on a wing margin caused by a heterozygous deletion of a gene located on the X chromosome that was consequently named “*NOTCH*” [13]. Many years later an initial description of Notch structure and functions was provided by Wharton and colleagues and it was shown to be a conserved intracellular pathway involved in a variety of cellular processes [14]. Despite the simplicity of its activation, a fine regulation of Notch signaling is required to avoid pathological effects including cancer development. Four Notch receptors (Notch1, Notch2, Notch3, Notch4) have been identified in humans. These receptors transduce signals by interacting with transmembrane ligands of the Delta-like (DLL1, 3 and 4) and Jagged (Jagged1 and 2) protein family on neighboring cells [15]. Once processed in the Endoplasmic Reticulum and in the Golgi, Notch receptors migrate to the cell membrane where they are activated by ligand interaction [16,17]. Upon ligand binding, the Notch receptor is cleaved by the protease ADAM10 or by TACE. The resulting fragment, called Notch extracellular truncation (NEXT), is then cleaved by γ-secretase and the Notch Intra Cellular Domain (NICD) is translocated to the nucleus allowing the transcription of Notch target genes including the Hairy enhancer of split homologs’ transcription factors (Hes and Hey) [18,19] (Figure 1). Low levels of NICD are sufficient to activate transcription because it acts as a transcriptional coactivator on the RBP-JK factor constantly bound to the promoter of target genes [20]. The activation of Notch signaling is finely regulated in different tissues driving organ morphogenesis during development [21].

Notch receptors are heterodimeric cell membrane proteins containing an extracellular subunit and a fragment that includes a transmembrane and an intracellular domain. Upon binding with ligand expressed on the surface of neighboring cells, two proteolytic cleavages occur. The first one takes place outside the trans-membrane domain by metalloproteinase TACE/ADAM10. The resultant Notch fragment, called Notch extracellular truncation (NEXT), is necessary for the second cleavage performed by γ-secretase within the trans-membrane region. This last proteolytical event releases Notch intracellular domain (NICD) that translocates to the nucleus, interacts with DNA binding proteins CSL (CBF1 Suppressor of Hairless Lag1) otherwise known as RBP-JK and Mastermind (MAML) transactivating target genes. 

### Notch Signaling in HCC

In the liver, hepatoblasts are the precursors of hepatocytes and cholangiocytes. Notch signaling can inhibit the differentiation of hepatoblasts into hepatocytes, allowing cholangiocyte formation [22,23]. Both the loss and gain of Notch functions may contribute to liver cancer development including both cholangiocarcinoma (CC) and HCC and different Notch receptors have different functions during liver cancer development [24]. Jagged1 plays a central role in the differentiation of hepatocyte progenitor cells (HPCs) and in hepatocyte proliferation during rat liver regeneration suggesting its possible involvement in HCC development [25,26]. Moreover, Jagged1 DNA copy number variation is associated with poor survival after liver cancer surgical resection [27] and its gene expression is comparable to Notch1 expression, suggesting an activation of Notch signaling that might be responsible for HCC development [27,28]. Occurrence of HCC in mice was reported after constitutive activation of NICD. Using bigenic the AFP-Notch Intracellular Domain (AFP-NICD) mice, in which Cre-mediated recombination in embryonic hepatoblasts results in the expression of a constitutively active form of Notch1 in >95% of cholangiocytes and hepatoblasts, Villanueva et al. showed HCC development in 100% of mice [29]. The analysis of differentially expressed genes revealed significant up-regulation of Notch target genes such as Hes1, and Hey2 and the activation of Notch signaling correlated with the transcriptional induction of insulin-like growth factor 2 (IGF2), an HCC promoter gene [30]. Aberrant Notch expression is frequently found in HCC tissue [31,32,33] where it participates in almost all the hallmarks of cancer, including resistance to anticancer treatments [33]. In line with this, several strategies inhibiting Notch increase treatment efficacy in preclinical models. Among these, γ-secretase inhibition (GSI) represents one the first approaches tested, including in clinical trials [34]. GSIs are a class of small molecules able to prevent the proteolytic cleavage of Notch receptors and the subsequent release of the Notch intracellular domain (NICD), which results from ligand interaction. Remarkably, gamma secretases are membrane-bound proteases involved in the cleavage of several other substrates including the beta-amyloid precursor, which accounts for their use in Alzheimer’s disease. Due to the pan-inhibition nature of GSIs, adverse effects have been described in clinical trials on advanced tumors, such as gastro-intestinal side effects, resulting from the blocking of the high constitutive expression of Notch in the GI tract. The poor results obtained in clinical trials in terms of tumor shrinkage or overall-survival (OS) might be related both to the absence of molecularly-based inclusion criteria and to the blockade of all four Notch receptors irrespective of their ligands. Indeed, experimental evidence shows that each Notch receptor is involved in specific and different functions. In addition, the downstream effects of Notch activation are strongly influenced by the interaction with specific ligands. Thus, alternative strategies have been considered, such as monoclonal antibodies targeting single Notch receptors or specific ligands. Many reviews have already summarized the current knowledge of Notch signaling in HCC development and have outlined the therapeutic potential of targeting Notch signaling in HCC [35,36,37]. Importantly, Notch plays pleomorphic roles in HCC, which relies both on the aberrant expression of specific Notch receptors and on their activation by specific ligands. A precise knowledge on the deregulated expression of each Notch receptor and ligand, coupled with phenotypic changes, is still lacking in HCC. This hampers the development of molecularly-targeted approaches. Notwithstanding, given the relevance of Notch aberrant activation in HCC, also in terms of resistance to treatments, this pathway is very attractive when hypothesizing combined treatment. Thus, in this review we highlighted the mechanisms involved in Notch signaling pathway regulation which might deserve attention when planning therapeutic strategies modulating Notch.

## 3. Notch and Hepatitis Viruses

**Figure 2:**
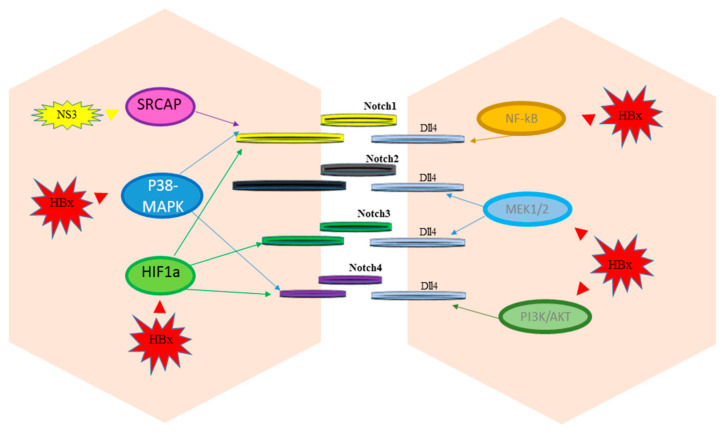
Notch signaling regulation by hepatitis viruses.

Hepatitis B virus (HBV) is a DNA virus that infects hepatocytes, and replicates by reverse transcription of a terminally redundant viral RNA. Infection of the liver may be either transient (<6 months) or chronic, depending on the ability of the host immune response to clear the infection. Upon infection, the virion DNA is converted in the nucleus to a circular DNA that assembles into a minichromosome, the template for viral mRNA synthesis. HBV encodes for seven proteins, preCore, core, pol, X (HBx), and the three envelope proteins. HBx has been reported as an important viral protein in the carcinogenesis and progression of HBV-associated HCC. HBx interacts with transcription factors in the nucleus such as nuclear factor-kappaB (NF-κB), activator protein 1 (AP-1), and cAMP response element-binding protein (CREB). HBx also interferes with various signal transduction pathways including Ras-Raf mitogen-activated protein kinase (MAPK), Janus kinase (JAK)/signaling transducer and activator of transcription (STAT), the Wnt/β-catenin pathway and phosphoinositide-3-kinase-protein kinase B/Akt pathway (PI3K-PKB/Akt) [38]. These pathways are involved in important cellular functions including cell proliferation and apoptosis. Therefore, it is not surprising that HBx is involved in malignant transformation even though the molecular mechanisms underlying this process are not fully elucidated. Notch signaling has been shown to play an oncogenic role in HCC and many studies have investigated its relationship with HBx. The expression of cytoplasmic Notch1 and nuclear Notch4 was upregulated by HBx in HepG2X cells and the upregulation of Notch1 by HBx was mediated by the p38 MAPK pathway [39]. Immunoistochemical staining showed a significant correlation between HBx and cytoplasmic Notch1 or nuclear Notch4, whereas other Notch receptors and HBx did not show any relationship. HepG2 and Bel-7404 cells transfected with HBx expression plasmids showed elevated Notch1, Notch3 and Notch4 mRNA and protein levels suggesting that HBx and HIF-1α may be responsible for the overexpression of Notch genes [40]. It was shown that HBx induces Notch1 activation and malignant transformation. Specifically, Notch1 exerts its effect on HBx-related HCC primarily via activation of the Wnt pathway proving that Wnt signaling is downstream of the Notch pathway in regulating proliferation of L02/HBx cells [41]. Using specific inhibitors, it has been found that MEK1/2, PI3K/AKT and NF-κB pathways are critical for HBx-mediated Dll4 upregulation and Notch1 cleavage. Silencing of HBx decreased the levels of Dll4 and cleaved Notch1 in HepG2.2.15 cells, increasing apoptosis and inducing cell cycle arrest, suggesting an important role of the HBx–Dll4–Notch1 axis in regulating cell survival in hepatocarcinoma [42]. These findings should be considered when planning future trials targeting Notch in HCC because, in active HBV infection, selected Notch receptors and ligands are expected to play a more relevant role and thus, their inhibition is expected to increase the response to treatment. Concerning HCV, the NS3 protein was found to activate the Notch transcriptional complex by binding to the proteins Snf2-related CBP activator protein (SCARP) and p400, thus inducing HES-1 expression [43] (Figure 2). In line with this, patients with chronic HCV infection displayed NOTCH1 up-regulation [44] which was also confirmed in vitro showing that both NOTCH1 and JAG2 are specifically induced following HCV infection of HuH7.5 cells [45]. Remarkably, several studies indicate also a relevant role for the Notch signaling in the modulation of the immune response in patients with HCV infection [46]. Interestingly, the Notch signaling blockade was demonstrated to inhibit the suppressive function of Tregs in patients with chronic HCV infection. In these patients, HCV clearance reduced both Notch1 and Notch2 expression as well as Tregs and Th17 proportions [47]. These findings, even though not obtained in HCC patients, provide a link between Notch signaling and immunotolerance which might deserve attention in HCC patients too. It is still unclear the extent to which Notch might influence immunotolerance in patients with chronic liver disease caused by other risk factors such as NASH. These data might provide further elements helping selection of patients for single or combined approaches targeting Notch and/or the immune response.

Proteins encoded by HBV (HBx) and by HCV (NS3) activate different cellular pathways that mediate the up-regulation of Notch receptors and ligands (Dll4).

## 4. Crosstalk of Notch Signaling with Other Signaling Pathways in HCC

**Figure 3:**
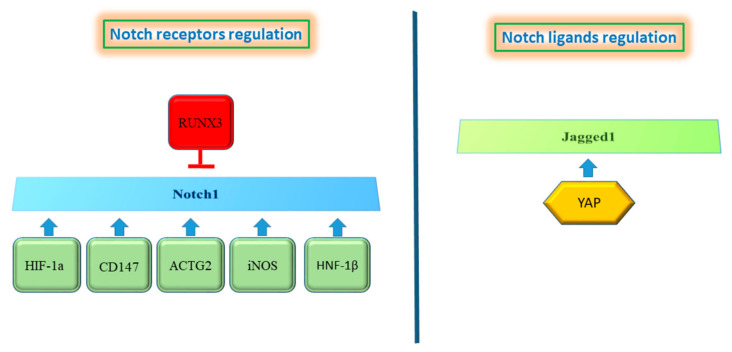
Proteins regulating the Notch pathway. the left panel shows proteins that induce Notch1 up-regulation, whereas Runx3 results in Notch1 inhibition. Only YAP protein is described to regulate Notch ligands (right panel).

Aberrant Notch signaling in HCC results from the activation of multiple molecules and pathways that dictate cellular processes, which ultimately drive the overexpression of Notch receptor and/or ligands. Herein we will report the most relevant triggers of Notch activation in HCC (Figure 3). HCC is frequently characterized by rapid growth of tumor cells that scavenge a substantial amount of oxygen, producing a hypoxic microenvironment that influences tumor aggressiveness and therapeutic response. In HCC the overexpression of the transcription factor named “hypoxia-inducible factors” (HIF) is a common genetic lesion in HCC. HIF is mainly involved in neo-angiogenesis, immune escape and in remodeling the activity of cancer stem cells [48]. In turn, HIF-1α increased the expression of Notch1, Notch2, Notch3 and Notch4 at both the mRNA and protein levels in HepG2 and Bel-7404 cell lines. Multiple HIF-1α binding sites were identified in the promoter regions of Noth1-4 suggesting that HIF-1α may enhance the expression of Notch1-4 through binding to the hypoxia responsive elements (CGTG) in their promoter regions [40]. Acting on Notch signaling, HIF-1α is a crucial regulator of epithelial-to-mesenchymal transition (EMT) and many studies focused on targeting HIFs in HCC confirmed reduced levels of Notch expression [49,50]. Similarly, the intracellular domain of the transmembrane glycoprotein CD147 (ICDs) regulates Notch1 expression by direct binding to the NOTCH1 promoter and induces the activation of the Notch signaling pathway resulting in cancer cell proliferation via Notch1 signaling. Accordingly, patients with a high nuclear CD147ICD expression display poorer overall survival compared with patients with a low nuclear CD147ICD expression, and repressing CD147 might represent a novel strategy to inhibit Notch1 signaling in HCC [51]. Runt-related transcription factor 3 (RUNX3) is a tumor suppressor gene whose expression is decreased in HCC, preventing apoptosis in HCC cells. Ectopic RUNX3 expression inhibits Notch signaling by decreasing jagged-1 (JAG1) mRNA in HCC. Based on the morphological changes in an HCC cell line with ectopic RUNX3 expression, it was hypothesized that RUNX3 increases cell–cell adhesion. Accordingly, the loss of RUNX3 protein resulted in an EMT-like change via increased expression of JAG1 [52]. Moreover, RUNX3 has been reported as a tumor suppressor that regulates HCC migration and invasion by both targeting the miR-186/E-cadherin/EMT axis and the Notch signaling pathway in HCC cell lines [53]. Specifically, RUNX3 interacts with the Notch1 intracellular domain preventing the RBP-J recognition motif on downstream gene promoters of Notch signaling [54]. Since aberrant methylation is the main reason of RUNX3 inactivation in cancer, reversal of DNA methylation by demethylating agents should restore RUNX3 activation preventing transcription of Notch target genes. Inducible nitric oxide synthase (iNOS) produces sustained nitric oxide (NO) concentrations in response to proinflammatory agents. Indeed, NO is a key mediator of chronic inflammation and participates in tumorigenesis affecting DNA repair, survival, cell proliferation, migration and angiogenesis. Mounting evidence highlights the role of iNOS in HCC development even though its functional interactions with biological pathways are not fully understood [55]. Recently, it has been demonstrated that iNOS activates Notch1 signaling through TACE/ADAM17 in CD24^+^CD133^+^ cells that possess stemness characteristics. This activation of Notch signaling accelerated HCC initiation and tumor formation in mice. Accordingly, CD24, CD133 and cleaved Notch receptors in human HCC were correlated with worse clinical outcome usually associated to cancer metastatization [56]. It might be hypothesized that pan-iNOS inhibitors would decrease HCC development and progression by interfering with Notch1 signaling. Understanding the cellular mechanisms that modulate the metastatization process is necessary to developing effective cancer therapies.

Actin proteins are involved in multiple intracellular processes including cell motility and maintenance of the cytoskeleton structure. Among them, actin gamma smooth muscle 2 (ACTG2) is over-expressed in HCC and is associated with poor prognosis and with a more aggressive phenotype, representing a promising therapeutic target in HCC metastasis. The use of shRNA to knockdown ACTG2 reduced cell migration and invasion in vitro and its silencing resulted in complete inhibition of metastasis in vivo. In line with this, ACTG2 overexpression significantly enforced HCC cells migration and metastasis. In order to understand cellular pathways guiding ACTG2-mediated metastasis, a gene expression analysis was performed in control and ACTG2-depleted SMMC-7721 cells. A panel of 27 genes involved in the regulation of metastasis were analyzed identifying Notch1 as the most down-regulated gene. Western blot analysis confirmed that Notch1 down-regulation in ACTG2 knockdown cells occurred also at the protein level. To confirm the role of Notch1 in ACTG2-mediated HCC metastasis, Notch1 over-expression was performed in ACTG2 silenced cells. In this setting, Notch1 restored the impaired invasion and migration of HCC cells ablated for ACTG2 [57]. Thus, it was confirmed that ACTG2 plays a critical role in HCC metastatization in a Notch1-dependent manner, representing a possible therapeutic target for HCC treatment. Hepatocyte nuclear factors (HNFs) are transcription factors expressed predominately in the liver where they orchestrate development and hepatocyte differentiation. Among these HNFs, hepatocyte nuclear factor-1beta (HNF-1β) is expressed in the liver progenitor’s cells and plays an important role in hepatobilary specification of hepatoblasts to the cholangiocytes lineage. In the adult liver, HNF-1β is expressed in the biliary compartment and in periportal hepatocytes. HNF-1β binds to DNA as a homodimer or heterodimer with HNF-1α and activates genes transcription [58]. Recent studies have shown that HNF-1β expression is associated with an increased risk of HCC development and progression. Immunohistochemistry analyses confirmed the association between high HNF-1β expression in HCC tissue and a significantly poorer disease-free survival (DFS). Accordingly, invasion ability of HCC cells overexpressing HNF-1β resulted higher than in control and these cells displayed an increased expression of hepatic progenitor cell (HPC) markers (CK7, CK19, CD133 and SOX9). A mechanistic study demonstrated that the up-regulation of these HPC markers was mediated by Notch signaling activation that plays a key role downstream of HNF-1β. Indeed, HNF-1β expression promoted the de-differentiation of HCC cells into liver cancer stem cells through the activation of the Notch signaling pathway. As a further proof, Notch inhibition by γ-secretase or shRNA to silence Notch1 in HNF-1β overexpressing cells, resulted in down-regulation of HPC markers, emphasizing the role of Notch1 as a driver of the progenitor phenotype [59]. Even though interfering with HNF-1β should represent a good strategy in HCC, modulation of transcription factors is still complicated and requires future investigation.

Yes-associated protein (YAP) is an oncoprotein located in the cytoplasm in an inactive form. Upon activation it translocates to the nucleus and, as an oncoprotein, it controls transcription factors that regulate cell proliferation [60]. Immunohistochemical analyses revealed weak staining for nuclear or cytoplasmic YAP in normal and cirrhotic livers. Conversely, elevated nuclear levels of YAP were observed in HCC, suggesting an association between YAP expression/localization and HCC development [61]. Accordingly, YAP inhibition in human HCC cell lines reduced cell viability, migration and invasion. Searching for a molecular mechanism mediating YAP functions in HCC, a transcriptomic analysis performed in YAP knockdown cells found Jagged1 as a YAP target gene. YAP knockdown in Huh7 cells reduced jagged1 protein levels leading to diminished Notch1 intracellular domain. The expression of Notch target gene Hes-1 was also affected by YAP knockdown attesting a reduced Notch activity. In line with this evidence, hepatocytes that overexpress YAP protein phosphorylated at serine 127, up-regulated Jag-1, leading to Notch pathway activation and increased proliferation. Jagged1 induction was mediated by binding of YAP to its transcriptional partner TEA domain family member 4 (TEAD4). The YAP/TEAD complex directly binds to the Jagged1 promoter, activating its transcription. However, interaction of YAP and TEAD4 occurs only in the presence of YAP protein phosphorylation at serine 127, suggesting the importance of this modification for YAP activity. To assess whether in vitro findings reflected the biology of human HCC, YAP, Jagged1 and Notch1 expression were analyzed by immunohistochemistry and a positive correlation was found among these proteins and their upregulation associated with a poor prognosis [62]. Inhibiting YAP/TEAD is an option for HCC therapy, however their unstructured nature renders them difficult to target. As an example Verteporfin (VP), showing the ability to disrupt the YAP–TED interaction in mouse models, also induces YAP independent effects preventing its clinical application [63,64]. Taken together, these findings outline the malignant phenotype in HCC, with particular regard to progenitor-like features and to the migration capability of cancer cells. However, the specific role of each one of the four Notch receptors, as well as their ligands, needs to be further elucidated and organized in a comprehensive evaluation in different HCC subgroups. This might provide the background for proposing specific Notch receptors and/or ligands as druggable molecules in selected patients.

## 5. Regulation of Notch Signaling by Non-Coding RNAs in HCC

Protein-coding genes represent less than 2% of the whole genome, suggesting a high representativeness of non-coding genes in the human transcriptome. Based on their length, non-coding transcripts can be dividend in small non-coding RNAs (< 200 bp) and long non-coding RNAs (from to 200 bp to 100 kb) [65]. In the last 20 years, a class of small non-coding RNAs (19–22 bp) called microRNAs (miRNAs), emerged as key regulators of gene expression in most of physio-pathologic conditions and their aberrant expression has been reported in solid tumors [66], including HCC [67,68,69]. MiRNAs act as post-transcriptional negative regulators by inducing mRNA degradation or inhibiting mRNA translation depending on miRNAs complementarity to their binding site in the 3′ untranslated regions (3′UTRs) of targeted mRNAs. In particular, perfect complementarity between the miRNA/mRNA sequence will lead to mRNA deterioration similarly to what is observed when small interfering RNA (siRNA) technology is employed, whereas imperfect complementarity will cause the interference of the mRNA translation machinery, resulting in decreased targeted protein expression with unchanged mRNA levels [70]. The identification of miRNA target genes is of utmost importance to characterize their biologic functions and implication in tumor development and progression. Many studies demonstrated a miRNA-dependent regulation of multiple molecules and downstream pathways with a central role in tumor progression, metastasis and drug resistance in HCC [67,71,72,73,74,75]. Strikingly, several studies on miRNA-based therapeutic strategies reported their safety and efficacy in preclinical models [76,77,78,79,80], and clinical trials showed their therapeutic potential in human diseases [81,82]. *TP53* is frequently mutated in human cancers and its mutations affect 25–30% of HCC cases [83]. *TP53* and miRNAs establish a complex network of mutual regulation and feedback loops in HCC leading to cell cycle regulation, apoptosis and sensitization to treatments [84]. We reported a positive feedback loop between the oncomiR-221 and *TP53* through the identification of *MDM2* among its target genes, leading to p53 activation and doxorubicin sensitization in HepG2 cells [85]. Interestingly, we observed that Notch3 silencing sensitized HCC cells to doxorubicin through p53 activation and identified a complex network of mutual regulations resulting in a Notch3/CG1/p53/miR-221/MDM2 axis promoting p53-sustained activation [31,86]. These findings highlight the regulation of common targets by HCC-specific miRNAs and Notch3 in HCC preclinical models, leading to the identification of new therapeutic strategies and patient stratification based on defined genetic backgrounds. Interestingly, miR-221 silencing with antagonist molecules “antagomiR-221” could be a promising therapeutic option for the treatment of p53-mutated HCCs where Notch3 silencing would be ineffective on p53 activation. A transgenic (TG) mouse model clearly demonstrated the causative role of miR-221 in hepatocarcinogenesis and represented an ideal tool to assess the efficacy of a chemically modified “antagomiR-221” strategy in limiting cancer progression in chemically-induced HCCs [80] and in preventing tumorigenesis in cirrhosis-associated liver tumors [79]. The upregulation of the cell cycle inhibitor, CDKN1C/p57, is one of the mechanisms underlying the anti-tumor activity of antagomiR-221 in the TG model and, intriguingly, it is a Notch3 target molecule [87], further highlighting the inter-relationships between Notch and HCC-specific miRNAs and providing the rationale for an miRNA-based approach in HCC to complement other Notch-targeted strategies.

Epithelial-to-mesenchymal transition (EMT) plays a central role in cancer progression and metastasis [88,89]. Several studies demonstrated the huge involvement of miRNAs and Notch signaling [90,91,92,93] in promoting this event, leading to increased aggressiveness and poor survival [94,95]. We observed that both miR-199a-3p overexpression and Notch1 silencing were responsible for the reduced invasion capabilities of HCC cells and, strikingly, a negative correlation was reported between miR-199a-3p and both Notch1 and E-cadherin in HCC patients [71,96]. We proved that miR-199a-3p negatively regulates E-cadherin expression through Notch1 direct targeting and that miR-199a-5p, deriving from the same precursor miRNA, correlates with miR-199a-3p and contributes to E-cadherin deregulation through its direct inhibition [73]. MiR-199a-3p is highly expressed in healthy liver, while it is downregulated in more than 70% of HCCs. Conversely, Notch1 expression is observed in tumor tissue only [96,97]. These data suggest that miR-199a-3p restoration, showing an anti-tumor effectiveness comparable to that of sorafenib in the TG221 preclinical model, might favorably modulate the Notch pathway too [98]. Remarkably, miR-34a represents a tumor suppressor miRNA in several solid malignancies and, for this reason, the first in-human clinical trial in oncology was designed using a liposomal miR-34a (MRX34) replacement strategy in patients with drug-refractory advanced primary tumors, including HCC. Despite promising dose-escalating results in the phase I study with dexamethasone pre-medication [99], the phase IIa clinical trial was terminated early due to severe adverse events (AEs) experienced in four patients associated with immune-related toxicity that seem not to be associated with the liposomal carrier but rather than to the RNA content [100]. Due to possible role for double-stranded RNA in triggering strong innate immune response, the amount of RNA per single dose might be a key point, together with chemical modification of oligonucleotides and delivery vehicles. Therefore, although exciting findings for miRNA-associated clinical trials in non-cancer diseases have been reported [81], proving their safety and efficacy, the design of clinical trials with this class of small RNA molecules requires further improvement in order to avoid severe AEs in oncologic patients. Notably, multiple miRNAs aberrantly expressed in HCC determine Notch1 signaling activation through different molecular mechanisms. Interestingly, mir-3188 is induced by the HBx protein in a TG mouse model and is overexpressed in HBV-related HCCs. CRISP specific knock-out (KO) of miR-3188 significantly reduced migration and invasion of HepG2 and SMMC7721 cells inactivating the Notch1 signaling pathway by direct targeting of the ZHX2 tumor suppressor gene [101]. MiR-449a inhibits migration and invasion by regulating EMT proteins via binding to Notch1 3′UTR in SMMC-7721 and HCCML3 cell lines. Accordingly, HCC cells with low expression of miR-449a are highly invasive and lead to recurrence within 6 months after surgery [102]. MiR-137 inhibits in vitro migration and invasion of HCC cells and attenuates their EMT process repressing Notch1 and Survivin [103]. In line with this observation, Notch1 was shown to be expressed in the non-tumor liver of rats that developed HCC, while it was not expressed in the liver of rats not developing HCC, suggesting that Notch1 is involved in HCC onset and in the so-called “field effect” representing the carcinogenic microenvironment assumed to cause accumulated genetic hits inducing cellular transformation [96,104]. Notch1 expression correlated with the expression of biomarkers of epithelial to mesenchymal transition in tumor specimens and in in-vitro models and with HCC metastasis [96,105,106]. Moreover, Notch1 expression in cirrhotic tissues was higher in patients with low (<2 years) versus high (>2 years) recurrence free survival, suggesting a role of Notch1 in the increased risk of HCC recurrence [96]. Overexpression of miR-3163 significantly inhibited the expression of ADAM-17 in the cytoplasm of MHCC97-H and LM-3 cells and, consequently, reduced the accumulation of NICD in the nucleus and enhanced the sensitivity of HCC cells to molecular targeted agents such as sorafenib [107]. In agreement with this study, the role of Notch3 in sorafenib resistance in vitro and in animal models was previously described. Specifically, Notch3 inhibition enhanced the apoptotic effect of sorafenib in HCC cells via specific down-regulation of p21 and up-regulation of pGSK3βSer9 [108]. An interesting study by Jung et al. reported the involvement of the miR-148a/IKKα/NUMB/NOTCH pathway in hepatocyte differentiation and suggested miR-148a mimics or NOTCH silencing as attractive chemo-preventive or therapeutic strategies, decreasing tumor grade malignancy, liver fibrosis and improving liver function [109]. Indeed, miR-148a is a potent inducer of hepatic differentiation in mice and humans and its down-regulation in HCC defines a cancer stem cell-like aggressive subtype [110]. Intra-peritoneal administration of a miR-148a-liposomal formulation in *PTEN* KO mice, before or after tumor development, decreased tumor size and incidence, tumor grade (hepatocellular and cholangiocellular adenomas were observed, instead of HCCs), and progenitor cell markers, while increasing hepato-specific markers (HNF4A, ALB and miR-122). In addition, IKKα targeting by miR-148a led to NOTCH signaling inactivation as determined by decreased HES1 and HEY1 expression levels in HepaRG cells, employed as an in vitro model to assess hepatocyte differentiation. NOTCH2 is the highest expressed isoform in liver progenitor cells [111]. Its overexpression reverted miR-148a phenotype in HepaRG cells; on the contrary, NOTCH inhibition by RO4929097 displayed both an anti-cancer and chemo-preventive activity in the same animal model. These findings are of particular relevance in the context of differentiation-targeted therapy that considers cellular differentiation as a plastic process. Bidirectional shifts from a stem to a differentiated state, and vice versa, might be of utmost importance in the onset of HCC especially in highly regenerative conditions, such as NASH and liver cirrhosis. Targeting self-renewal or differentiation capacities of tumor-initiating cells might be indeed a promising option in both therapeutic and preventive perspectives. NOTCH2 was also shown as a mediator of anti-miR-21 therapeutic strategy in *PTEN* KO mice and HepaRG progenitor cells [111]. MiR-21 is upregulated in almost all solid tumors [66], including hepatocellular and cholangiocellular carcinomas [75,112]. In the animal model, miR-21 was up-regulated in tumors with respect to surrounding livers but, interestingly, its expression was limited to peritumoral areas, neoplastic biliary cells and ductular reaction regions and co-expressed in osteopontin (OPN) positive progenitor cells. In situ hybridization confirmed a similar expression pattern in human HCCs. Anti-miR-21 treatment triggered a dramatic change in tumor morphologic characteristics leading to a decrease in cholangiocarcinoma (CCA) and hepatocholangiocellular carcinoma (HCA) and improved tumor grade showing an increase in well differentiated cells with respect to pleomorphic and heterogeneous HCCs. In vitro experiments demonstrated that NOTCH2 is the most expressed isoform in HepaRG cells and its inhibition by anti-miR-21 treatment was responsible for reduced OPN expression and apoptotic cell death of CD24-positive progenitor cells. NOTCH2 is the only isoform to decrease in mice tumors treated with anti-miR-21. Due to its well-known role in hepatoblast differentiation towards the biliary cell lineage [113] and its involvement in liver carcinogenesis [29,114], NOTCH inhibition might account for decreased tumor malignancy following miR-21 silencing in the mouse model. Because of its influence on survival of tumor-initiating cells and expansion of tumor-associated stromal cells, miR-21 targeting might hold promise as a therapeutic option for liver cancer prevention in at-risk populations. Indeed, the lack of biomarkers represents a limiting step in the diagnosis of early HCC. Due their high stability in body fluids, circulating miRNAs are promising diagnostic and prognostic tools in liver diseases [115], with miR-21 being one of the most abundant miRNAs detected in the circulation showing a good diagnostic value for early HCC diagnosis [116]. An outstanding study described an entangled network between the circ-CDYL/miR-892a/HDGF/miR-328-3p/HIF1AN axis and both the PI3K/AKT and NOTCH2/SURVIVIN downstream pathways in very early HCC. Specifically, circ-chromodomain Y like (CDYL) was the most upregulated circular RNA in early HCCs and contributed to derepression of competing endogenous RNAs (ceRNAs) hepatoma-derived growth factor (HDGF) and hypoxia-inducible factor asparagine hydroxylase (HIF1AN), by sponging and sequestering miR-892a and miR-238-3p, respectively. Overexpression of circ-CDYL in HCC cell lines and xenograft models increased cell proliferation, colony and sphere formation capabilities, stem cell markers, chemoresistance and tumorigenesis. In particular, HDGF upregulation led to the activation of its specific receptor, Nucleolin, which triggered the activation of the PI3K/AKT/mTOR and β-catenin/MYC proliferation pathways, whereas HIF1AN upregulation determined an increase in the stemness marker SURVIVIN by blocking the internalization of NOTCH2 intracellular motif NICD. Finally, circ-CDYL combined with HDGF and HIF1AN performed better than AFP in predicting HCC at early but not advanced stages, holding promise as a biomarker for the surveillance of early HCCs. This study demonstrated that, at very early stages, NOTCH2 might behave as a TS gene contributing to the expression of molecules such as SURVIVIN and c-MYC, involved in the survival of tumor-initiating cells [117]. The dual role of miRNAs [118,119] and NOTCH signaling [120,121] is indeed a common event in cancer, further increasing biologic complexity and emphasizing the need for accurate preclinical models and bioinformatics tools aiding the translation of experimental findings into clinics.

**Figure 4:**
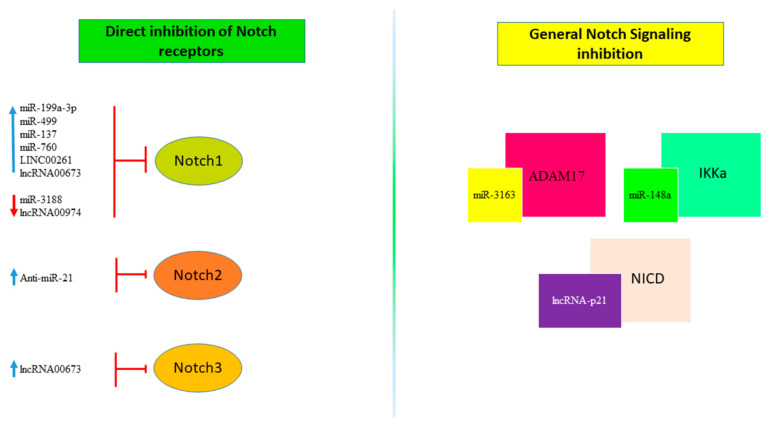
Non-coding RNAs regulating Notch Signaling. non-coding RNAs, including miRNAs and lncRNAs, target Notch signaling through mechanisms that directly regulate the expression of one of the Notch receptors (left panel) or by inhibition of proteins involved in the Notch signaling pathway (right panel). The red arrow indicates down-regulation while the blue one indicates up-regulation.

Another RNA class gaining importance in the epigenetic regulation of cancer related pathways is represented by long non-coding RNA (lncRNA), RNA molecules that are more than 200 bases in length. LncRNAs are involved in epigenetic modification of DNA, and regulation of transcriptional and post-transcriptional gene expression [122]. Usually, these non-coding RNAs possess secondary structures to interact with DNA, RNA and proteins. Long non-coding RNAs have cell-specific expression and their transcription responds to specific stimuli. Dysregulation of lncRNAs’ expression influences cell proliferation, angiogenesis, resistance to apoptosis, and evasion of tumor suppressors [123]. Recent findings have identified hundreds of lncRNAs that play crucial roles in the regulation of HCC development and progression [124,125]. Functional assays demonstrated that overexpression of LINC00261 inhibited cell proliferation, invasion and EMT process in vitro, by downregulating Notch1 and Hes-1 expression in HCC cells [126]. Chen et al. showed that lncRNA00673 interference resulted in cell cycle arrest in the G0-G1 phase and increased apoptosis. Moreover, cellular invasion, evaluated by transwell experiments, was inhibited by lncRNA00673 interference. In vivo experiments confirmed that decreased lncRNA00673 significantly inhibited tumor formation ability of HCC cells through Notch1 and Notch3 down-regulation [127]. LincRNA-p21 was initially identified as a direct transcriptional target of p53 acting as a suppressor of translation by binding target mRNA [128]. The lincRNA-p21 expression is downregulated in both HCC tissue and HepG2 and SMMC-7721 cells. Overexpression of lincRNA-p21 inhibited Notch singling and EMT, while its downregulation led to the opposite result. In more detail, in SMMC-7721 cells transfected with pcDNA-lincRNA-p21, Notch signal-related proteins Hes-1 and NICD were downregulated and invasion of HCC cells was inhibited. In line, the invasion capability of HepG2 cells was promoted upon si-lincRNA-p21 transfection and the Notch signaling inhibitor DAPT reversed this effect [129]. These findings outline the complex network of regulatory events involving Notch signaling and different classes of ncRNAs, which are selectively deregulated in specific cancer types and participate to Notch aberrant expression. Their identification might help to hypothesize novel treatment strategies aimed at hinting at aberrant regulatory mechanisms limiting the re-activation of redundant pathways due to their multi-targeting nature (Figure 4). Despite very encouraging preclinical results, the translation of ncRNAs findings into clinics needs caution. The identification of suitable patient subgroups and the optimization of oligonucleotide formulations and delivery systems are central points for the design of future clinical trials in HCC and the most important preclinical studies are reported in Table 1.

## 6. Concluding Remarks

The Notch signaling pathway is an evolutionary conserved pathway that regulates key cellular processes. Its deregulated activation is a hallmark of cancer; hence, targeting Notch activity has been proven an effective approach in different human cancers including HCC. Indeed, the development of a Notch targeted therapy appears a rational strategy to modulate a crucial signaling for cancer cells. Notch-directed therapies include the unselective γ-secretase inhibitors or monoclonal antibodies (mAbs) that instead inhibit specific Notch receptors and ligands, either alone or combination with other traditional approaches [141]. However, a few important hurdles still limit the direct translation of promising preclinical work into patient benefit. Some functional limitations of monoclonal antibodies emerged, such as tissue accessibility, inadequate pharmacokinetics as well as impaired interactions with the immune system resulting in treatment failure. Many studies have highlighted the need of increasing tissue penetration to improve the outcome of antibody therapy [142,143] and that is probably the reason why tarextumab did not show benefit over the placebo in lung cancer (NCT01859741). Similarly, further clinical developments of anti-DLL-4 monoclonal antibody demcizumab were halted due to the lack of benefit over the normal standard of care. A study on cetuximab and trastuzumab in xenograft models showed that their tumor distribution could be enhanced with increased dose; however, hypoxic areas inside the tumors remain difficult reach [144]. Reaching optimal drug concentrations in tumor tissue represents a significant challenge in cancer therapy. In addition, therapeutic antibodies have to compete with patients’ IgGs for binding to specific epitopes [145]. On the other hand, gamma-secretase inhibitors were tested in different human cancers and poorly tolerated overall. Indeed, the majority of patients suffered from gastrointestinal toxicity, due to the simultaneous blockade of Notch1 and Notch2 receptors and to the inhibition of Notch signaling in non-tumor tissues. Moreover, while different Notch receptors are co-expressed in tumors, they do not mediate the same effect, further compromising the therapeutic efficacy of pan-Notch inhibitors. Finally, GSIs might impair the antitumor immune response since CD8 T cells require Notch signaling for the expression of canonical effector molecules including granzyme B and interferon-γ [146]. Accordingly, lower levels of Notch-1/2 in murine tumor-infiltrating CD8 T cells have been reported after GSIs treatment and correlate with reduced tumor control [147]. As an alternative approach, it was also hypothesized to inhibit Notch transcription factors; however, limitations associated with their delivery are difficult to overcome [148]. However, the pan-notch inhibitor CB-103 (Cellestia Biotech AG) that interrupts the assembly of the Notch transcription complex on DNA, leading to down-regulation of Notch target genes, showed in vitro efficacy and is now in clinical trials for human solid tumors (NCT034226790). Intracellular trafficking is a main regulator of cell signaling since the majority of receptors are transmembrane or membrane-associated proteins and Notch signaling is coupled to intracellular trafficking. Interfering with these regulatory mechanisms might represent an innovative way to block Notch signaling in cancer. Several factors including ion concentration, control Notch trafficking. Accordingly, recent in vitro studies showed that Notch trafficking can be impaired through the disruption of zinc homeostasis resulting in the suppression of the Notch target gene Deltex E3 Ubiquitin Ligase 1 (DTX1) and consequently the NVS-ZP7-4 zinc transporter inhibitor (ZIP7) induced apoptosis in T-ALL, warranting further investigation [149]. Other possible approaches to inhibit Notch signaling might be acting on the post-translational modifications required by Notch for trafficking or by acting on the endosomes that transport these receptors [150,151]. Finally, targeting molecules achieving Notch signaling in tissue cancer only, in order to obtain a more selective approach than that offered by targeting γ-secretases or Notch itself, might be considered. As an example, ncRNAs, which proved to be important regulators of Notch signaling in cancer cells, might be targeted to control the aberrant activation of Notch as well as of other oncogenic pathways at the same time [152]. Of course, a careful selection of ncRNA selectively deregulated in cancer tissue should be performed. In this regard, many studies have been performed in mouse models by using double-stranded RNA-mediated interference (RNAi) and single-stranded antisense oligonucleotides (ASOs). For example, inhibiting MALAT1 with ASO suppresses metastasis in mice bearing breast cancer and in lung cancer xenograft models [153,154]. Based on the results obtained in preclinical models, several studies on ncRNA-guided precision medicine have been conducted also in HCC (Table 1). A better understanding of the mechanisms driving Notch signaling activation in HCC tissue, together with a characterization of HCC molecular subgroups, will contribute to develop therapeutic strategies enhancing the efficacy of treatments.

## Figures and Tables

**Table 1:** Important long noncoding RNA in hepatocellular carcinoma (HCC).

Name	Size (bp)	Deregulation in HCC	Role in HCC	Ref
**MALAT1**	8708	Upregulated	Tumor metastasis and recurrence	[130,131,132]
**HOTAIR**	12649	Upregulated	Associated with invasion	[130,133]
**HULC**	1638	Upregulated	Associated with tumor growth	[131,134]
**HOTTIP**	6839	Upregulated	Associated with tumor progression	[135,136]
**H19**	2660	Upregulated	Promotes cell proliferation	[137,138]
**UCA1**	7375	Upregulated	Associated with disease outcome	[139,140]
